# 1. Actinomycosis, and 2, Strongylus Gigas in Dogs

**Published:** 1900-07

**Authors:** F. Torrance

**Affiliations:** Winnipeg, Canada


### REPORTS OF CASES.

### 1. ACTINOMYCOSIS, AND 2, STRONGYLUS GIGAS IN DOGS.

By F. Torrance, D.V.S.,
WINNIPEG, CANADA.

1. Actinomycosis. A pure-bred and well-broken pointer was brought to me suffering from ascites. Examination of the heart and liver proved negative, but the urine was found to be albuminous. Medical treatment failed entirely to relieve the dropsy, and I had recourse to tapping, and removed in this way a large quantity of fluid. The abdomen very speedily filled up again and the operation had to be repeated frequently. The amount of fluid removed was often very great, and on one occasion measured 160 fluidounces. The dog had a good appetite all through his illness, but kept losing flesh continuously until he was reduced to skin and bone.

The owner now consented to his destruction, which was accomplished painlessly by the use of chloroform. The post-mortem examination followed immediately. On opening the abdomen a large quantity of clear fluid escaped. The peritoneum was healthy except for the presence of a few flakes of adherent lymph, but on the surface of the intestines the blood-vessels stood mapped out as distinctly as in an injected specimen. The liver was greatly engorged with blood, which flowed from it when incised like water from a sponge. The kidneys appeared healthy on section, but when examined microscopically one of them was found to be affected with interstitial nephritis.

In the thorax a large mass was seen occupying the region between heart and diaphragm, and involving the posterior part of the right lung, part of the pericardial sac, and the diaphragm. The mass was as large as a cricket-ball and quite solid. On section the cut surface had a marbled appearance and resembled carcinoma. Recognizing the rarity of a malignant tumor in such situation, I removed it *en masse* and sent it to Dr. Bell, the provincial bacteriologist, for his opinion. He reported finding actinomycetes in the growth, and was of the opinion that they were the cause of it. Other small nodules of the disease could be found in the lung, and it is probable that invasion took place through the respiratory tract.

Actinomycosis in the dog must be a rare disease, for there is no mention of it in such works as Friedburger and Fröhner's *Pathology*, nor have I ever seen it described in any veterinary journal. In this case it caused ascites by interfering mechanically with the return of blood from the abdomen through the vena cava.

2. STRONGYLUS GIGAS. A husky dog which had been bought from the Indians living upon the shores of Lake Winnipeg was brought to this city with others for shipment to the Yukon district, where dog teams are the chief mode of locomotion in the winter. While chained up with his comrades in a large loft he was found one morning lying almost dead in a pool of blood.

At first it was supposed that another dog had got loose and had bitten him, accidentally severing an artery. However, a careful examination failed to reveal any wound, but discovered the fact that the blood was escaping from the urethra. The dog died before any veterinary aid could be rendered, and a post-mortem examination was made at once.

On opening the abdomen the organs appeared healthy, with the exception of the bladder, which contained blood, and the right kidney. The capsule of this kidney was of a livid color, and the outline of the organ was irregular. On cutting into it there was hardly any kidney tissue, nothing but a thin layer next the capsule. All the rest of the organ was converted into a large sac, in which was snugly coiled up an enormous worm as thick as a lead-pencil. When straightened out the parasite measured eighteen inches, and, no doubt, while making his breakfast on kidney, had bitten through the renal artery, causing the death of his host and his own transfer to a specimen-bottle.

This case may throw some light upon the source from which this parasite is derived. It has been suggested that perhaps this is also a parasite of fish, and in support of this view is the fact that the Indian dogs in the vicinity of the big lakes are usually fed on fish, which are thrown to them whole and uncooked.

